# Motivations, challenges, and benefits of first aid knowledge popularization volunteerism among undergraduate medical students: a qualitative study

**DOI:** 10.3389/fpubh.2025.1701431

**Published:** 2025-12-31

**Authors:** Ruiyu Huang, Siyu Li, Yanxia Guo, Xiaofang Yang, Baolu Zhang

**Affiliations:** 1 School of Continuing Education, Guiyang Healthcare Vocational University, Guiyang, China; 2 School of Nursing, Southwest Medical University, Luzhou, China; 3 Faculty of Nursing and Midwifery, Jiangsu College of Nursing, Huaian, China; 4 Guiyang Maternal and Child Health Care Hospital, Guiyang, China

**Keywords:** first aid knowledge popularization, volunteer service, medical students, qualitative study, expectancy-value theory

## Abstract

**Background:**

First aid knowledge popularization volunteerism provides medical students with opportunities to develop practical skills. However, there is currently limited attention to First aid knowledge popularization volunteerism, especially regarding the experiences of volunteers themselves. Therefore, our study explored the motivation, challenges, and benefits of undergraduate medical students participating in first aid knowledge popularization volunteerism based on the expectancy-value theory.

**Methods:**

This qualitative descriptive study, guided by Expectancy-Value Theory, explored the motivations, challenges, and benefits of undergraduate medical students participating in first aid knowledge popularization volunteerism. We conducted 27 semi-structured interviews with first aid knowledge popularization volunteerism team members recruited from a medical university in Sichuan Province, China. Theoretical saturation was achieved through iterative data collection and analysis, with the final sample size determined when no new themes emerged from additional interviews. Data were analyzed using Braun and Clarke's thematic analysis approach.

**Results:**

Our study identified three main themes with eleven subthemes. Motivations included voluntary spirit, self-achievement, reward mechanism, and peer influence. Challenges comprised skill deficiencies, psychological challenges, objective constraints, and time constraints. Benefits encompassed psychological benefits, incentives, and skill enhancement.

**Conclusion:**

Our study research results indicate that the motivation of undergraduate students in first aid knowledge popularization volunteerism stems from the dual effects of internal drive and external motivation. The challenges they face mainly come from the influence of internal obstacles and external limitations, while the ultimate benefits are reflected in both psychological growth and ability improvement. Universities should develop specialized first aid education curricula integrating emergency simulation scenarios and establish FAKPV-specific peer mentorship programs to enhance motivation, provide specialized training combining first aid techniques with community-specific public education pedagogy to address challenges, and create systematic opportunities for volunteers to witness direct impact through follow-up assessments and real-time feedback to maximize benefits.

## 1 Introduction

First aid knowledge and skills play a critical role in public health. Research demonstrates that correct first aid treatment can reduce mortality by 1.8%−4.5% for trauma events, while immediate bystander intervention in cardiac arrest cases can substantially improve survival outcomes (1–3). However, significant gaps persist in public first aid literacy worldwide, and the public's lack of first aid knowledge can lead to serious consequences during the crucial minutes before professional medical help arrives (4). First Aid Knowledge Popularization Volunteerism (FAKPV) represents a specialized form of health education volunteer activities, distinct from general medical volunteerism in its focus on preventive education and community capacity building (5, 6). FAKPV can enhance the public's first aid skills through popularizing first aid knowledge (5), thereby empowering community members to provide life-saving interventions during emergencies. Despite widespread first aid skill insufficiency (4), FAKPV provides a mechanism for addressing this public health gap through trained volunteers.

Medical students possess medical training and proximity to clinical knowledge, positioning them as potential first aid educators (7, 8). Research has demonstrated that medical student volunteers can serve hundreds of patients with multiple identified needs, providing students with enhanced understanding of the holistic nature of patient care while developing skills to build patient relationships and understand psychosocial factors shaping health outcomes (9). During the COVID-19 pandemic, 66.13% of medical students globally expressed willingness to volunteer (10). However, despite this willingness and potential, significant limitations exist in medical students' emergency preparedness. A study in India showed that 67.8% of medical students surveyed had no training in emergency medicine or public health crisis management (11). This gap between potential and preparedness underscores the importance of understanding how medical students develop as first aid educators through volunteer experiences.

Volunteering is any activity in which time is given freely to benefit another person, group, or organization (12). Within medical education contexts, volunteer service has been recognized as a key indicator of students' overall competence, with some universities incorporating it into their training systems as practical credits, rating volunteers based on their service hours (13). Medical student volunteerism represents an important intersection of professional development and public service, providing educational opportunities including early exposure to patient interaction, social determinants of health, and interdisciplinary collaboration (9, 14). Prior research has established several key findings about volunteer experiences. Studies have documented that volunteering positively impacts volunteers' social, mental, and physical health and well-being (15–17). Research has found that medical students' motivations for volunteering include altruistic intentions to help patients, practical learning opportunities to apply medical knowledge, and social responsibility to serve community health needs (18–20). Studies have also identified that challenges affect volunteer retention rates and service integration effectiveness (21, 22), while benefits enhance participation willingness (23).

Despite the recognized role of FAKPV in public health and medical students as volunteer educators, significant gaps remain in understanding the comprehensive volunteer experience within this specialized context. Specifically, limited knowledge exists about the interplay between students' motivations to volunteer, the challenges they encounter, and the benefits they derive from FAKPV participation. Three important limitations characterize existing research. First, studies focus primarily on service recipients rather than volunteers themselves. Second, research examines individual dimensions in isolation, typically focusing on single aspects such as motivation, challenges, or benefits, rather than comprehensive experiences. Third, integrated theoretical approaches that can capture the dynamic interactions between motivations, challenges, and outcomes remain underutilized in volunteer research, particularly in specialized health education contexts like FAKPV.

Theoretical frameworks that can capture these dynamic interactions remain underutilized in volunteer research. While frameworks such as Clary and Snyder's Volunteer Functions Inventory and Ryan and Deci's Self-Determination Theory have contributed foundational insights into volunteer motivation (24, 25), additional theoretical perspectives may be needed to comprehensively examine the dynamic interplay between motivations, challenges, and outcomes in specialized health education contexts. The Expectancy-Value Theory (EVT) is a well-established motivational framework that examines how individuals' beliefs about their ability to succeed and the importance they place on the task influence their engagement and performance (26, 27). Unlike the Volunteer Functions Inventory's focus on functional motivations or Self-Determination Theory's emphasis on basic psychological needs, EVT's dual dimensions of success expectancy and task value allow for comprehensive examination of both motivational drivers and outcome perceptions within volunteer experiences. EVT has been widely applied in educational contexts to understand learning motivation and healthcare settings to explore career attractiveness and student engagement with clinical learning and community service activities (28–31). Unlike motivational frameworks that focus primarily on functional categories or psychological needs, EVT's integration of cognitive beliefs about ability and subjective task value provides a unified lens for understanding the complete volunteer experience arc from initial motivation through challenge navigation to benefit realization. Therefore, our study explores the motivations, challenges, and benefits of FAKPV among undergraduate medical students based on EVT.

## 2 Methods

### 2.1 Study design

Our study employed the Qualitative Descriptive (QD) method to explore medical students' experiences, motivations, challenges, and gains in FAKPV emergency science education volunteering. This method offers flexibility in obtaining rich data and deeply understanding phenomena (32–34). We conducted 27 semi-structured interviews with FAKPV team members from a university in Sichuan Province, China. Using Braun and Clarke's thematic analysis framework (35, 36), all interviews were recorded, transcribed verbatim, and systematically analyzed. This flexible analytical approach enabled a comprehensive understanding of our data. This study is grounded in an interpretivist paradigm, recognizing that participants' experiences are socially constructed through their interactions within FAKPV contexts. We adopted the qualitative descriptive approach because it allows comprehensive exploration of participants' perspectives without imposing pre-existing theoretical categories while remaining open to theoretical insights that emerge from the data. To ensure comprehensive and transparent reporting of our qualitative research, this study adheres to the Consolidated Criteria for Reporting Qualitative Research (COREQ) checklist (37).

### 2.2 Expectancy-value theory

Our study is guided by the theoretical framework of EVT, which indicates that behavioral choices are influenced by two main determinants: expected success, which encompasses individuals' confidence in their ability to perform tasks and achieve desired outcomes, and task value, which reflects the perceived importance, utility, and intrinsic worth of engaging in specific activities (26, 27). While established frameworks such as Clary and Snyder's Volunteer Functions Inventory and Ryan and Deci's Self-Determination Theory have provided valuable insights into volunteer motivation (24, 25), EVT offers distinct advantages for analyzing FAKPV experiences (26, 27). Unlike the Volunteer Functions Inventory's functional categorization or the Self-Determination Theory's focus on basic psychological needs, EVT's dual-component structure allows for simultaneous examination of both expectancy beliefs and value perceptions within volunteer contexts. This framework is particularly suitable for understanding specialized volunteer activities like FAKPV, where both technical competence expectations and perceived value of health education outcomes influence participation decisions.

**Figure 1:**
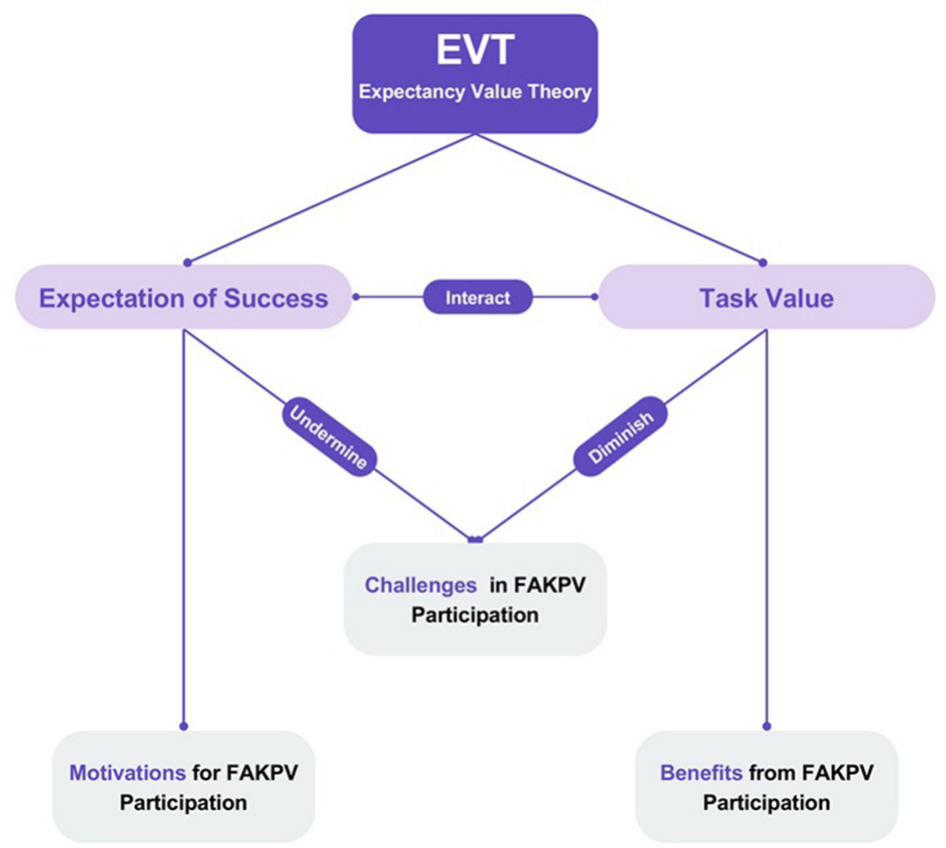
EVT - framework analysis of motivations, challenges, and benefits in undergraduate medical students' FAKPV participation.

In our study, expected success directly corresponds to students' motivations, manifesting as their self-efficacy and confidence regarding their capacity to engage effectively in first aid knowledge popularization activities. On the other hand, task value encompasses the perceived importance, utility, and personal significance that individuals attribute to FAKPV. In our study, task value represents the benefits that students derive from their volunteer participation, including the positive outcomes, meaningful experiences, and perceived value they gain. Challenges serve as a critical link between these two dimensions, as they can simultaneously undermine expected success by diminishing students' confidence while also reducing task value by creating barriers to meaningful engagement and positive outcomes, as illustrated in Figure 1.

In our study, we designed semi-structured interview guidelines based on EVT, focusing on motivations (expectancy beliefs), challenges (expectancy-undermining and value-reducing factors), and benefits (value-enhancing outcomes). During the thematic analysis phase, we used the EVT theoretical framework as a guide to systematically identify and extract themes related to motivations, challenges, and benefits. While our study is grounded in EVT principles, we maintained an open attitude during data analysis, accepted that themes outside the theory emerged.

### 2.3 Participants

Our study was conducted at a medical university in Sichuan Province, China. All recruited students had participated in FAKPV to ensure that they could provide meaningful insights based on direct experience.

The inclusion criteria were as follows: (1) current enrollment as medical students at a university in Sichuan Province, China, (2) primary participation in at least two emergency science popularization volunteer activities, and (3) voluntary participation. We excluded participants who (1) had incomplete participation records in emergency science popularization volunteer activities, (2) were unable to effectively express personal thoughts and experiences due to language barriers or cognitive impairments, and (3) exhibited significant emotional instability or psychological stress during interviews that raised ethical concerns.

We adopted the purposeful sampling method. The authorized staff refers to the administrative coordinators and student leaders of the university's FAKPV volunteer organization who maintain participant records and have direct knowledge of students' volunteer engagement levels. These staff members initially screened potential participants based on our inclusion criteria, specifically identifying students who had participated in at least two FAKPV activities and had complete participation records. The research team then contacted eligible students via phone or WeChat to explain the study purpose and invite participation. All contacted students who met the eligibility criteria agreed to participate and provided informed verbal consent, resulting in a final sample of 27 participants. No participants refused participation or dropped out after enrollment. At the same time, data collection and preliminary analysis were conducted. We evaluated the emergence of new topics after every 4–5 interviews. The final sample size of 27 participants was determined through theoretical saturation, achieved when data collection no longer yielded new themes or insights relevant to our research questions. This approach aligns with Guest et al., who demonstrated through systematic analysis of 60 interviews that data saturation typically occurs by the 12th interview in relatively homogeneous samples, while our larger sample size of 27 enhances the methodological rigor and credibility of findings (38).

### 2.4 Researcher positionality

The researcher A, who is in charge of all the interview work, is an undergraduate student with experience in volunteer services. Researchers B and C respectively have backgrounds in quantitative research methods and medical education. The team's familiarity with the FAKPV environment helps establish relationships and understand the viewpoints of the participants, but we also realize that our positive attitude toward volunteer education might have an impact on our interpretation. To address this issue, throughout the analysis process, researchers maintained reflexive awareness of their positions as medical educators and documented potential influences of their professional backgrounds on data interpretation through analytic memos.

### 2.5 Data collection

To collect data, Researcher A conducted in-depth semi-structured face-to-face interviews from July to October 2023 in private, quiet settings to ensure participant comfort and confidentiality. All interviews were conducted by Researcher A to maintain consistency and minimize interviewer-related bias across data collection. All interviews were recorded and transcribed verbatim, then cross-checked with the original recordings to ensure accuracy. During each interview, Researcher A maintained field notes documenting key observations and non-verbal cues, while Researcher B served as a dedicated note-taker, recording detailed conversational content, key responses from the participants, contextual information, and reflective notes on interview question adequacy, participant comprehension issues, and potential modifications needed for subsequent interviews. This dual documentation approach ensured comprehensive data capture and enhanced the reliability of qualitative data collection. Data analysis began concurrently with data collection in August 2023, allowing the research team to refine interview probes based on emerging themes.

During the interview, participants shared their motivations, challenges, and benefits of participating in FAKPV. The questions in the interview guide were as follows:

1. What motivated you to participate in FAKPV?
2. Could you describe the most significant challenges you've encountered while volunteering?
3. How do you usually approach and resolve these challenges?
4. What personal gains have you experienced through participating in FAKPV?

Probing questions and follow-up prompts were used flexibly throughout interviews to explore participants' responses in greater depth. The complete interview guide is provided in Supplementary File 2.

Each interview was assigned a unique code to protect participant privacy, and all personal identifiers were removed during transcription and analysis. Participants were informed of their right to withdraw from the study at any time.

### 2.6 Data analysis

**Figure 2:**
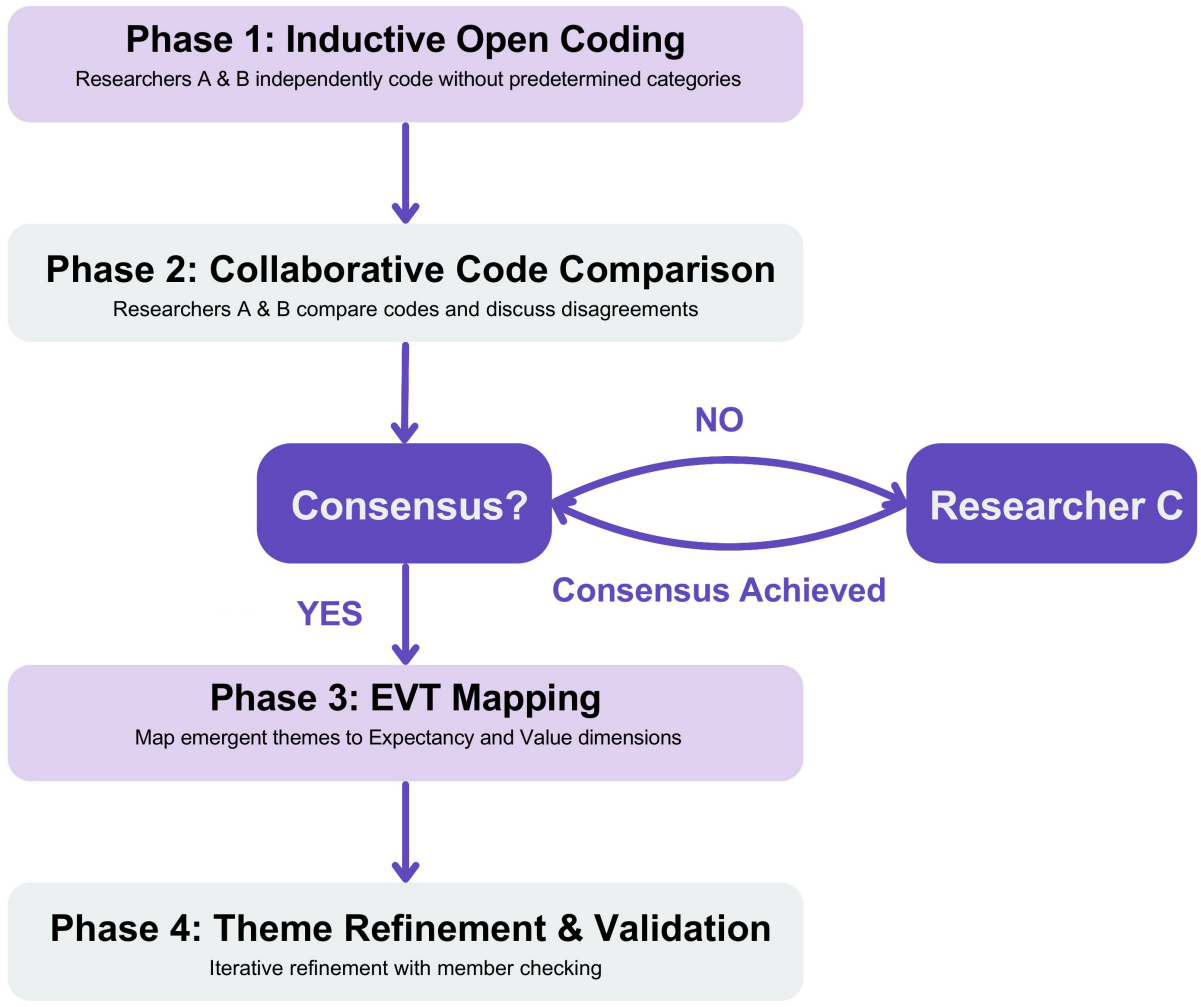
Coding process and consensus resolution.

Our study employed Braun and Clarke's thematic analysis framework (45, 46), systematically integrated with EVT. We adopted a hybrid approach combining inductive and deductive coding strategies. Initially, we conducted open inductive coding to allow themes to emerge naturally from the data without imposing predetermined theoretical categories. Following this inductive phase, we systematically mapped the emergent themes to EVT constructs, using the framework's expectancy and value dimensions as an analytical lens to examine the relationships between motivations, challenges, and benefits in Figure 2. First, all recorded interviews were independently transcribed verbatim by both Researchers A and B, with each researcher transcribing the complete dataset. The transcriptions were then cross-verified, where Researcher A validated Researcher B's transcripts and vice versa, ensuring accuracy and consistency across all materials. To achieve comprehensive data familiarization, Researchers A and B repeatedly read the transcripts and made initial observational notes, capturing initial observations and emerging concepts. During this phase, researchers focused on open reading and observation, approaching the data without predetermined theoretical constraints while concentrating on the content presented in the data itself and participants' original expressions. Second, Researchers A and B independently conducted line-by-line systematic coding of the transcripts, generating additional codes while remaining sensitive to EVT constructs. Specifically, codes related to motivations were categorized as expectancy-related elements such as self-efficacy and confidence, or value-related elements, such as perceived importance and personal significance. Challenge-related codes were analyzed for their impact on both expectancy and value dimensions, while benefit-related codes were mapped to value enhancement outcomes within the EVT framework. Following the initial coding phase, they compared their codes, identifying areas of overlap while forming and continuously refining the coding scheme. Figure 2 illustrates the complete coding process and consensus resolution mechanism, while Supplementary File 1 provides representative examples of how raw interview data were systematically transformed into codes, categories, and themes. This triangulation approach involved multiple researchers independently coding the same data, comparing interpretations, and reaching consensus through collaborative discussion, thereby reducing individual researcher bias and enhancing analytical rigor. Third, building on the established coding framework, themes and subthemes were developed by systematically grouping related codes, allowing thematic structures to emerge naturally from the data. Through iterative discussions, Researchers A, B, and C collaboratively constructed themes and subthemes, ensuring thematic frameworks captured participants' experiences while remaining grounded in the data.

The research team observed substantial alignment between emerging themes and EVT constructs, leading to the adoption of EVT conceptual language to refine thematic labels while preserving the authentic meaning of participant data. While EVT provided a valuable theoretical lens, the research team remained open to emergent themes that extended beyond the theoretical framework, ensuring that participant experiences were not constrained by predetermined theoretical boundaries. Subsequently, to ensure thematic coherence, comprehensive thematic refinement was conducted through a systematic review process. Researchers A and B examined the coherence of coded data within themes and subthemes and evaluated individual thematic elements' relationship to the entire dataset and research questions, thereby constructing a thematic framework that is grounded in the original data while incorporating perspectives from the EVT theoretical framework. Throughout the study, data collection and preliminary analysis were conducted iteratively, with saturation assessment occurring after every 4–5 interviews. Theoretical saturation was systematically assessed using multiple criteria established by Guest et al.: (1) no new themes emerged from additional interviews, (2) existing themes were well-developed and comprehensive, and (3) further data collection yielded only repetitive information without contributing new insights to the research questions. The research team collaboratively evaluated saturation after every 4–5 interviews during both data collection phases. Final saturation was confirmed when the last 5 interviews produced no new thematic content, and all major theme categories had reached sufficient depth and breadth. This systematic approach to saturation assessment following established methodological guidelines and ensured that our sample size of 27 was adequate for achieving comprehensive thematic development while maintaining methodological rigor. Finally, the research team proceeded with further defining and refining thematic nomenclature. The ultimate thematic definitions and naming both considered the language of the original data and drew on EVT theoretical terminology, while also accommodating insights that emerged beyond the theoretical scope, forming an organic integration of theory and data. To validate the interpretations, three original participants reviewed the identified themes and subthemes and confirmed whether these findings accurately reflected their FAKPV volunteer experiences. Throughout the analysis process, researchers maintained reflexive awareness of their positions as medical educators and documented potential influences of their professional backgrounds on data interpretation through analytic memos.

### 2.7 Trustworthiness

When coding disagreements arose, Researchers A and B first attempted to reach consensus through discussion. If disagreement persisted, Researcher C reviewed the disputed codes and facilitated resolution through collaborative deliberation until consensus was achieved. All disagreements and their resolutions were documented in our audit trail to maintain transparency and methodological rigor. This process implementing investigator triangulation to enhance the credibility and reliability of the analysis (39, 40).

### 2.8 Ethical considerations

Our study involved human participants and received ethical approval from Southwest Medical University's Biomedical Ethics Committee (SWMUIRBTX-202406-0022). All participants provided informed verbal consent via phone or WeChat prior to participation, which was audio-recorded and documented. Verbal consent was approved by the ethics committee as appropriate for this minimal-risk qualitative interview study. Participants were assured of anonymity through coded identifiers and informed of their voluntary participation with the right to withdraw at any time without consequence. Research data remained strictly confidential and accessible only to the research team. All audio recordings were transcribed within 24 h and securely stored with restricted access to the research team only. De-identified transcripts were used for all analysis. Audio files will be destroyed after study completion in accordance with institutional policies.

## 3 Results

Twenty-seven undergraduate medical students, aged 19–22, participated in our study. They represented five medical specialties, predominantly clinical medicine, with 2–11 instances of volunteer service participation. Table 1 presents an overview of individual participant characteristics, while Table 2 provides a demographic summary. Interviews lasted between 26 and 54 min.

**Table 1:** Demographic characteristics of participants (*N* = 27).

***P***	**Age**	**Major**	**Gender**	**Volunteer participation times**
P1	22	Chinese and Western Medicine Clinical Medicine	Female	3
P2	20	Pediatrics	Female	5
P3	20	Medical Image Science	Female	5
P4	22	Clinical Medicine	Male	11
P5	22	Chinese and Western Medicine Clinical Medicine	Female	4
P6	20	Clinical Medicine	Female	2
P7	22	Medical Image Science	Female	6
P8	19	Clinical Medicine	Female	6
P9	21	Traditional Chinese Medicine	Female	6
P10	19	Traditional Chinese Medicine	Female	2
P11	21	Clinical Medicine	Female	3
P12	20	Clinical Medicine	Male	2
P13	19	Clinical Medicine	Female	6
P14	20	Chinese and Western Medicine Clinical Medicine	Female	7
P15	23	Clinical Medicine	Male	8
P16	20	Clinical Medicine	Female	4
P17	19	Traditional Chinese Medicine	Female	3
P18	21	Pediatrics	Female	7
P19	22	Clinical Medicine	Male	5
P20	20	Chinese and Western Medicine Clinical Medicine	Female	6
P21	20	Medical Image Science	Female	2
P22	21	Clinical Medicine	Female	8
P23	20	Traditional Chinese Medicine	Female	4
P24	22	Clinical Medicine	Male	6
P25	19	Chinese and Western Medicine Clinical Medicine	Female	3
P26	20	Traditional Chinese Medicine	Female	5
P27	20	Clinical Medicine	Female	7

**Table 2:** Demographic characteristics summary (*N* = 27).

**Characteristic**	**Value**
Age (years), Mean ± SD	20.2 ± 1.6
**Gender distribution**, ***n*****(%)**	
Female	23 (85.2%)
Male	4 (14.8%)
Volunteer participation times, Mean ± SD	5.0 ± 2.5
**Major distribution**, ***n*****(%)**	
Clinical Medicine	13 (48.1%)
Chinese and Western Medicine Clinical Medicine	5 (18.5%)
Traditional Chinese Medicine	4 (14.8%)
Medical Image Science	3 (11.1%)
Pediatrics	2 (7.4%)

SD, standard deviation; *n*, number of participants; %, percentage.

Three main themes emerged from the analysis of interviews with participants: (1) Motivations, (2) Challenges, and (3) Benefits of FAKPV. Table 3 provides a comprehensive overview of themes, sub-themes, and categories.

**Table 3:** Themes, subthemes and categories obtained from students.

**Themes**	**Subthemes**	**Categories**
Motivations (*N* = 25,92%)	Voluntary spirit	Altruistic motivation
		Public health education drive
		Demonstrating professional ethics and social responsibility
	Self-achievement	Enhancing practical skills
		Improving communication abilities
		Boosting self-confidence
		Interdisciplinary knowledge exchange
	Reward mechanism	Academic credit and conduct point rewards
		Fulfillment of curriculum requirements
	Peer influence	Role model effect
		Group participation motivation
		Social network expansion
Challenges (*N* = 24,88%)	Skill deficiencies	Limited professional knowledge
		Inadequate practical skills
		Communication skill challenges
	Psychological challenges	Performance anxiety
		Fear of responsibility
		Self-doubt
	Objective constraints	Insufficient funding for activities
		Institutional barriers
	Time constraints	Balancing volunteer work with academic responsibilities
		Schedule disruptions
Benefits (*N* = 26,96%)	Psychological benefits	Sense of social responsibility
		Sense of psychological fulfillment
		Sense of self-achievement
		Team spirit
	Incentives	Academic achievements and recognition
		Professional development opportunities
		Family health benefits
	Skill enhancement	Enhancement of emergency care skills
		Enhancement of communication skills
		Practical experience accumulation

### 3.1 Motivations

The theme “Motivations” encompasses the driving forces that compel undergraduate medical students to engage in FAKPV. Based on the participants' experiences, this concept encompasses four key sub-themes: voluntary spirit, self-achievement, reward mechanism, and peer influence.

***Voluntary Spirit***, which represents participants' intrinsic motivation to serve others and contribute to society, consists of altruistic motivation, the desire to promote public health education, and the demonstration of professional ethics and social responsibility. During their participation in FAKPV, interviewees displayed enthusiasm for helping others, expressed the importance of disseminating health knowledge, and viewed these activities as opportunities to practice medical ethics. They regarded these activities as both fulfilling social responsibilities and embodying personal values.

“*I participate in FAKPV because... I genuinely want to help others.”* (P4)

“*I believe our role isn't just about treating future patients. We should also take on the role of health educators, spreading health knowledge… so that the public can save lives even before we arrive.”* (P14)

“*I feel that my motivation to participate in volunteering is influenced by our university motto (Virtue and expertise, benevolence and healing). I'm putting this concept into practice, contributing to society.”* (P15)

“*As a medical student, I believe we have a responsibility to share our knowledge with the community... FAKPV allows me to contribute beyond just studying.”* (P20)

Notably, P4's statement “I genuinely want to help others” demonstrates how altruistic intentions represent the intrinsic value dimension of EVT, where students perceive FAKPV as inherently worthwhile regardless of external rewards. Meanwhile, P20's emphasis on “a responsibility to share our knowledge with the community” reflects a sense of professional obligation intertwined with intrinsic motivation—this aligns with the same EVT framework, showing that the intrinsic value of FAKPV is also rooted in medical students' recognition of their social and professional roles.

***Self-achievement***, referring to personal growth and skill development through volunteer participation, consists of enhancing practical skills, improving communication abilities, interdisciplinary knowledge exchange, and boosting self-confidence. Beyond altruistic motivations, this sub-theme reveals how students also sought personal development opportunities through FAKPV participation. Through participating in FAKPV, interviewees practiced first aid techniques, interacted with diverse groups, and gained a sense of accomplishment from successfully conducting activities. They perceived these experiences as both beneficial to others and conducive to their comprehensive skill development.

“*I participate in FAKPV because I want to apply the first aid knowledge learned in class to practice... This can help me become more proficient in practical skills.”* (P4)

“*I feel my communication skills are not good enough... Participating in FAKPV allows me to interact with different people and learn how to explain medical knowledge. It (Doctor-patient communication) is important in future work.”* (P10)

“*Participating in FAKPV helps others, and seeing people appreciate my efforts... This feeling of being needed boosts my confidence.”* (P14)

“*Working with students from nursing and public health programs in FAKPV has broadened my perspective... I learned about community health approaches that complement our clinical training.”* (P18)

“*Collaborating with students from different medical specialties helps me understand how various disciplines approach emergency care... It's valuable interdisciplinary learning.”* (P24)

Specifically, P18's account reinforces the attainment value dimension of EVT, highlighting how FAKPV addresses both competence development needs and confers utility value for future professional practice.

***Reward Mechanisms***, which encompass external incentives and recognition systems that motivate participation, include academic credit and conduct point rewards, and fulfillment of curriculum requirements. While intrinsic motivations were primary, this sub-theme captures how external incentives also shaped students' participation decisions. Interviewees indicated that these external incentives enhanced their motivation to participate in FAKPV. They viewed these rewards as recognition of their efforts and beneficial to their academic development. While intrinsic motivation was primary, these reward mechanisms played a role in encouraging continued participation.

“*Participating in FAKPV offers credits, which helps with my course completion... It also improves my comprehensive quality score, which is quite important for future awards and honors.”* (P2)

“*In fact, another reason is that the university requires us to complete a certain number of volunteer service hours... FAKPV perfectly meets this requirement.”* (P8)

“*The credits are nice to have, but honestly, that's not why I keep coming back... I've already met my requirements, but I continue because I genuinely enjoy teaching first aid.”* (P14)

P14 articulates a shift from extrinsic to intrinsic motivation as students accumulate experience, showing how initial utility value can develop into more profound intrinsic value over time. However, participants with more volunteer experience (6–8 times) tended to de-emphasize reward mechanisms in their narratives, with several explicitly stating that credits became less important as they gained more experience, suggesting that accumulated experience may shift motivational emphasis from extrinsic to intrinsic factors.

***Peer influence***, describing the social factors that shape volunteer participation decisions, includes the role model effect, group participation motivation, and social network expansion. In addition to personal motivations and external incentives, this sub-theme reveals how social factors played an important role in students' decisions to join FAKPV. Interviewees described how classmates and friends shaped their decision to join FAKPV. They mentioned the positive impact of outstanding peers engaging in volunteer services, experiencing the atmosphere of collective participation, and recognizing these activities as opportunities to expand their social circles.

“*Seeing the excellent students in our class participating in FAKPV... I think they're doing the right thing, and I want to learn from them.”* (P5)

“*Several of us in the dorm joined the FAKPV together... It feels interesting to do this kind of thing as a group.”* (P7)

“*Participating in FAKPV allowed me to meet many students from other majors... This is beneficial for future study and work.”* (P12)

P5 highlights how peer modeling shapes perceived task value via social comparison, revealing that FAKPV participation offers social validation benefits beyond individual motivations. From an EVT perspective, voluntary spirit and self-achievement represent intrinsic and attainment value dimensions, while reward mechanisms reflect utility value providing tangible academic benefits. Peer influence demonstrates how task value can be shaped through social comparison and group dynamics.

### 3.2 Challenges

The theme “Challenges” encompasses the obstacles and difficulties that undergraduate medical students encounter during their engagement in FAKPV. This concept includes four sub-themes: skill deficiencies, psychological challenges, objective constraints, and time constraints.

***Skill deficiencies***, referring to gaps in knowledge and abilities that limit effective volunteer performance, include limited professional knowledge, inadequate practical skills, and communication skill challenges. Interviewees described experiencing capability gaps during FAKPV participation, noting difficulties in handling complex situations, performing practical operations, and communicating with diverse groups. These deficits presented significant barriers to their volunteer service.

“*When explaining first aid to the public, I encounter questions I'm not prepared for... I realize my knowledge isn't comprehensive enough, and there are some I really can't answer.”* (P1)

“*My practical training is still limited... some of my first aid actions aren't standard… I often need guidance from others to complete the whole process.”* (P10)

“*When explaining first aid knowledge to people of different age groups... sometimes I didn't know how to express it in a way they could easily understand.”* (P9)

P1 captures how perceived knowledge gaps directly erode expectancy for success, as the student expresses doubt about their capacity to act effectively as a first aid educator. However, some participants described skill deficiencies less frequently and with less intensity, suggesting that accumulated experience correlates with reduced perception of skill gaps and enhanced technical confidence.

***Psychological challenges***, encompassing the emotional and mental pressures experienced during volunteer activities, include performance anxiety, fear of responsibility, and self-doubt. Beyond technical capability gaps, this sub-theme captures how students also confronted emotional and psychological barriers during their volunteer work. Interviewees described the psychological pressures they experienced during FAKPV participation. They mentioned feeling nervous when demonstrating first aid skills in public, worrying about the responsibility of performing emergency aid, and doubting their abilities.

“*Every time I have to demonstrate first aid skills in front of a group, I get really nervous... I'm afraid of making mistakes and affecting others' learning.”* (P3)

“*Thinking about being responsible for saving someone in a real emergency... I feel I'm not fully competent yet, and this sense of responsibility puts a lot of pressure on me.”* (P9)

“*Sometimes I doubt whether I'm really capable of teaching first aid to others... I always feel like I haven't learned enough myself.”* (P15)

“*After my first few sessions, the nervousness mostly disappeared... Now I actually look forward to teaching because I've learned to prepare thoroughly and trust my training.”* (P20)

P3 demonstrates how performance anxiety diminishes expectancy by creating psychological barriers to confident teaching, even when students possess adequate technical knowledge. In contrast, P20 illustrates how successful experiences progressively enhance expectancy and reduce psychological costs, creating a positive feedback loop that sustains participation. Notably, participants with more than six volunteer experiences described reduced anxiety and greater confidence in teaching first aid, suggesting that more experience correlates with enhanced psychological resilience and self-efficacy. A small number of participants with extensive experience reported minimal psychological pressure, indicating they had developed effective coping mechanisms through repeated exposure.

***Objective constraints***, representing external barriers beyond participants' direct control, include insufficient funding for activities and institutional barriers. In addition to personal capability and psychological factors, this sub-theme reveals how students encountered structural barriers beyond their individual control. Interviewees described certain limitations they encountered while participating in FAKPV. These constraints affected the implementation of FAKPV activities.

“*The budget for FAKPV activities organized by our college is limited... We can only provide services to a few communities, unable to cover more people in need.”* (P10)

“*I found it challenging to collaborate with communities for FAKPV activities... Sometimes, communities change their plans at the last minute, which makes our preparation work very difficult.”* (P13)

Interestingly, the perception of objective constraints appeared relatively consistent across experience levels, suggesting these external barriers are systemic rather than experience-dependent.

***Time constraints***, describing difficulties in managing volunteer activities alongside other responsibilities, include balancing volunteer work with academic responsibilities and dealing with schedule disruptions. Alongside external constraints, this sub-theme captures how students also struggled with managing the competing demands of volunteer service and academic responsibilities. Interviewees expressed concerns about maintaining academic performance while actively participating in FAKPV. They also highlighted the need for flexibility in adapting to last-minute changes in volunteer activities.

“*Participating in FAKPV activities is meaningful, but sometimes it clashes with exam preparation... It's really difficult to find a balance between volunteer service and studying.”* (P12)

“*Sometimes we receive last-minute notices for FAKPV activities... Having to adjust my original plans suddenly often makes me feel overwhelmed.”* (P15)

“*I use a detailed schedule to plan both my studies and volunteer activities... With good time management, I've found I can balance both without much difficulty.”* (P18)

*This suggests that while time constraints represent a real cost in EVT terms, effective coping strategies can mitigate their impact on continued participation*.

P18 indicates that while time constraints constitute a tangible cost in the context of EVT, the adoption of effective coping strategies can alleviate their impact on sustained participation. However, some participants indicated they had developed effective time management strategies through experience, allowing them to better integrate volunteer activities with academic demands, though time pressure remained a consideration.

From an EVT perspective, skill deficiencies and psychological challenges directly impact the expectancy dimension by undermining students' confidence in their ability to succeed. The finding that these challenges diminish with experience suggests that accumulated practice enhances self-efficacy. Time constraints and objective constraints represent cost factors in EVT's cost-value dimension, constituting personal sacrifices and external barriers that students must weigh against perceived participation value.

### 3.3 Benefits

The theme “Benefits” captures the various ways undergraduate medical students find value in their engagement with FAKPV. This concept encompasses three key sub-themes: psychological benefits, incentives, and skill enhancement. These aspects reflect the diverse experiences and perceptions of students regarding the rewards of their volunteer work, spanning personal growth and professional development.

***Psychological benefits***, encompassing the emotional and mental rewards derived from volunteer participation, include a sense of social responsibility, psychological fulfillment, self-achievement, and team spirit. Interviewees noted an increased sense of social responsibility for their role in FAKPV, a sense of purpose from sharing vital knowledge, personal growth through overcoming challenges, and improved interpersonal skills through teamwork.

“*Participating in FAKPV made me realize that as a medical student, I have a responsibility to spread first aid knowledge to the public... It's not just learning, but a social responsibility.”* (P8)

“*When I see participants successfully mastering CPR skills, I feel very fulfilled... Knowing that the knowledge I've taught might save lives...”* (P10)

“*After successfully organizing a community first aid lecture, I felt a great sense of achievement... It made me believe I can contribute to society beyond just studying medicine.” (*P11)

“*During FAKPV activities, I learned how to work closely with other students... This teamwork experience is important for our future as doctors.”* (P13)

“*Seeing families apply the first aid skills we taught them gives me immense satisfaction... It reinforces my commitment to medical education.”* (P22)

P22's account highlights how witnessing tangible community impact delivers powerful emotional rewards, which in turn sustain long-term volunteer commitment among participants. Some participants described deeper and more nuanced psychological benefits, particularly emphasizing professional identity development and long-term commitment to health education, suggesting that accumulated experience intensifies the psychological rewards of volunteer participation.

***Incentives***, referring to tangible rewards and opportunities gained through volunteer participation, include academic achievements and recognition, professional development opportunities, and family health benefits. Beyond emotional satisfaction, this sub-theme reveals how students also gained tangible rewards that provided practical value for their academic and professional development. Interviewees described various motivating factors from participating in FAKPV, encompassing both personal growth and broader social impact.

“*Participating in FAKPV offers extra credits... This not only helps with my academic performance but also serves as an important reference for awards and honors.”* (P1)

“*Through FAKPV, I‘ve connected with some hospital experts... They've given me a lot of advice on future career development, which is very helpful for my career planning.”* (P6)

“*After learning first aid, I can apply it at home too... Once, when my younger brother was choking on food, I immediately used the Heimlich maneuver and actually saved him.”* (P15)

“*The credits and certificates are secondary for me... What really matters is knowing I'm making a difference in community health literacy.”* (P22)

However, some participants did not emphasize tangible incentives in their narratives, focusing instead on intrinsic rewards, suggesting individual differences in the salience of external vs. internal benefits.

***Skill enhancement***, representing the improvement of practical abilities and professional capabilities through volunteer experiences, includes improvement of emergency care skills, enhancement of communication abilities, and accumulation of practical experience. In addition to psychological and tangible rewards, this sub-theme captures how students experienced concrete improvements in their professional capabilities through repeated practice. Interviewees shared how FAKPV activities contributed to their professional development, noting improvements in their technical proficiency, interpersonal effectiveness, and clinical readiness.

“*Through repeated practice, my CPR skills have significantly improved... Now I feel I can respond more calmly in emergency situations.”* (P7)

“*Through FAKPV activities, my verbal expression skills have greatly improved... Now when explaining first aid knowledge to groups, I can express myself more fluently and confidently.”* (P8)

“*FAKPV provided me with many practical opportunities... These experiences have given me more confidence in future clinical work.”* (P13)

Participants with more volunteer experiences reported greater confidence in their enhanced skills and described more diverse skill improvements across technical, communication, and leadership domains, indicating that cumulative participation amplifies the breadth and depth of skill development benefits.

From an EVT perspective, psychological benefits embody intrinsic value through emotional satisfaction and professional identity development, while incentives reflect utility value by providing tangible academic and career advantages. Skill enhancement simultaneously reinforces both value perceptions through concrete personal growth and enhances expectancy for future success by increasing competence and confidence. The finding that benefits intensify with experience suggests a positive feedback loop where successful participation increases both perceived value and success expectancy.

## 4 Discussion

Our study identified the motivations, challenges, and benefits experienced by undergraduate medical students participating in FAKPV. This qualitative study delves into how these experiences affect students' participation in public health education and community service in the context of medical education.

### 4.1 Motivations

Our study revealed that medical students' motivations for FAKPV participation encompass voluntary spirit, self-achievement, reward mechanisms, and peer influence. From an EVT perspective, voluntary spirit and self-achievement represent intrinsic and attainment value dimensions, reflecting students' alignment between personal values and volunteer activities (26, 27). Voluntary spirit and self-achievement align with previous research demonstrating that altruistic intentions and personal growth consistently emerge as core motivational factors across diverse volunteer contexts (41–43). This consistency underscores the universal nature of intrinsic motivation in volunteer engagement and highlights the importance of fostering these fundamental drives in educational settings. Our study uniquely found reward mechanisms as a significant motivational factor. Within the EVT framework, academic credits and recognition serve as utility value, as they provide tangible benefits that directly support students' academic and professional goals (26, 27). Previous research has demonstrated that academic credits and honors can significantly increase volunteer participation rates (44), while values-based education effectively cultivates volunteer spirit and skill development enhances self-achievement in volunteer activities (45–47). A previous study examining students across the United States, Canada, and Finland has documented similar altruistic drives among student volunteers, suggesting that these motivational patterns demonstrate consistency across diverse cultural contexts (48). The dynamic interplay between expectancy and value components explains participation patterns observed in our data. Students who initially joined for academic credits but lacked teaching confidence frequently reported anxiety and considered discontinuation. However, those who persisted beyond initial challenges demonstrated a critical shift. As their expectancy increased through successful teaching experiences, they began to recognize higher attainment value. FAKPV aligned with their emerging professional identity. This positive feedback loop, where increased expectancy enhances perceived value, which in turn motivates further engagement despite costs, explains why experienced volunteers consistently reported greater intrinsic motivation than novices. Conversely, students who discontinued participation exhibited a different pattern. Persistent low expectancy combined with decreasing utility value led to a negative calculation where time investment outweighed perceived benefits. This illustrates expectancy-value theory's core premise. Motivation results not from value alone, but from the multiplicative relationship between expectancy and value (27). Universities should therefore develop specialized first aid education curricula that integrate emergency simulation scenarios and community-specific health needs assessment, fostering students' understanding of their critical role as health educators in emergency preparedness. Additionally, institutions should establish FAKPV-specific peer mentorship programs incorporating structured competency frameworks where experienced volunteers guide newcomers in developing confidence for public first aid instruction.

### 4.2 Challenges

Our study found four primary challenges that students encountered during FAKPV participation, including skill deficiencies, psychological challenges, objective constraints, and time constraints. From an EVT perspective, skill deficiencies and psychological challenges directly impact the expectancy dimension by undermining students' confidence in their ability to succeed in volunteer activities (26, 27). When students doubt their teaching competence or fear making errors in first aid instruction, their expectancy for success decreases, which can diminish motivation and engagement. This expectancy-based barrier operates through a specific psychological mechanism. When students perceive their first aid knowledge as inadequate, they anticipate negative outcomes such as teaching errors, community member confusion, and personal embarrassment. This reduces their expectation of success. According to expectancy-value theory, even when students recognize high attainment value and utility value, low expectancy acts as a bottleneck (27). If students believe they cannot succeed, high value cannot compensate. Our data support this pattern. Students with 2–3 volunteer experiences reported highest anxiety levels despite acknowledging FAKPV's importance, precisely because their expectancy had not yet developed through sufficient successful experiences. Skill deficiencies and time constraints correspond with previous studies identifying similar barriers including knowledge gaps and scheduling conflicts in volunteer contexts (49–51), which confirms that these obstacles represent fundamental challenges inherent in volunteer engagement across diverse service domains. Our study distinctively identified skill deficiencies as particularly prominent among medical students. According to the EVT framework, these skill deficiencies directly impact the expected success dimension by diminishing students' confidence in their ability to perform volunteer activities effectively (26, 27). Previous studies have indicated that structured training programs enhance students' practical capabilities and volunteer effectiveness (52, 53), while systematic approaches addressing skill gaps and scheduling flexibility improve volunteer retention rates (54). Research conducted across 68 countries has documented similar implementation challenges including cultural barriers, language difficulties, and skill preparation gaps, suggesting that these obstacles reflect universal patterns in volunteer programs globally (55). Medical schools should accordingly provide specialized FAKPV training programs that combine advanced first aid techniques with community-specific public education pedagogy, enabling students to teach diverse community populations. Furthermore, establishing partnerships with local emergency medical services can provide real-world emergency simulation experiences with immediate feedback mechanisms specifically designed for FAKPV volunteers.

### 4.3 Benefits

The benefits students derived from FAKPV participation included psychological benefits, incentives, and skill enhancement. Within the EVT framework, these benefits function as value reinforcement mechanisms that strengthen students' continued participation. Psychological benefits represent intrinsic value satisfaction, incentives provide utility value through tangible rewards, and skill enhancement contributes to both competence development and career-related utility value (26, 27). Importantly, these benefits also enhance students' expectancy for future success by building confidence through accumulated experience and demonstrated competence. These benefits create a success cycle that sustains motivation. Psychological benefits such as increased confidence and sense of contribution directly enhance expectancy for future volunteer activities. Students who felt satisfied after teaching believed they could succeed again. Simultaneously, skill enhancement increases both expectancy through greater competence and utility value through career-relevant capabilities (27). This dual reinforcement explains the resilience of experienced volunteers. They had developed sufficient expectancy-value momentum that occasional setbacks did not deter continued participation. In contrast, novice volunteers with marginal expectancy and primarily extrinsic value orientations were more vulnerable to discouragement from initial difficulties. Psychological benefits and skill enhancement align with extensive research documenting personal growth, professional development, and emotional satisfaction as consistent outcomes of volunteer participation (53, 56, 57), reinforcing the transformative potential of volunteer experiences and validating the multidimensional nature of volunteer benefits. Our study uniquely found incentives as a distinct benefit category. Within the EVT framework, these incentives represent tangible task value outcomes that reinforce students' participation decisions by providing concrete rewards for their efforts (26, 27). Previous studies have revealed that emphasizing psychological satisfaction and skill development opportunities effectively enhances volunteer recruitment and retention (45, 46), while highlighting professional development benefits strengthens volunteer engagement outcomes (45, 58). Previous research primarily from the United States has demonstrated that volunteering produces multidimensional benefits that extend beyond individual gains, suggesting that FAKPV outcomes may have broader applicability (16). FAKPV programs would benefit from creating structured opportunities for volunteers to witness the direct impact of their education efforts through systematic follow-up community assessments and real-time emergency response feedback from trained community members. Establishing FAKPV-specific competency recognition systems with measurable outcomes can acknowledge expertise in emergency education delivery and community health promotion.

Our study identified several findings that transcend the scope of conventional EVT frameworks. Regarding motivation, peer influence emerged as a crucial social factor where students joined FAKPV because classmates participated or roommates encouraged group involvement, creating a social contagion effect. EVT, while powerful in examining individual expectancy and value assessments, was developed primarily to understand individual decision-making processes (26, 27), which may explain why peer influence emerged as an additional factor in our study. Previous research has documented peer effects in participation behaviors (22) and demonstrated that social interactions play an important role in willingness to volunteer, which is more significant than the activity itself (59). In terms of challenges, emotional stress and fear of responsibility represent deeper emotional costs than the practical considerations EVT traditionally examines, suggesting that volunteer decision-making involves complex emotional dimensions beyond cognitive evaluations. These profound emotional responses to volunteer responsibilities extend beyond EVT's primary focus on cognitive cost-benefit calculations (26, 27), suggesting that affective dimensions warrant additional theoretical consideration in volunteer contexts. Studies have revealed significant psychological challenges and identified burnout syndrome among volunteers who experience exhaustion, cynicism, and feelings of inefficacy (60). Additionally, objective constraints including insufficient funding and institutional barriers operate at organizational levels outside EVT's emphasis on personal perceptions and choices. Previous research has identified organizational-level barriers and institutional constraints as major obstacles to volunteer participation (54, 61). Concerning benefits, family health outcomes emerged when students applied first aid skills to help family members during emergencies, extending beyond EVT's focus on individual gains to include unexpected social impacts. While EVT traditionally concentrates on individual-level outcomes (26, 27), our findings suggest that volunteer experiences may generate extended social and familial impacts that warrant further investigation. Research has demonstrated that volunteering produces multidimensional benefits that surpass individual gains (17, 47), with evidence showing that the benefits of health professional volunteering extend to the wider community and society (21). Previous studies have demonstrated that peer mentoring programs effectively improve psychosocial well-being and academic development among medical students (62), while psychological support services have proven effective in managing emotional challenges and providing crisis intervention (46). Based on these insights, we suggest that FAKPV programs should establish structured peer mentorship systems specifically designed for emergency education volunteers, provide accessible psychological support services to address unique emotional challenges related to emergency care responsibility, and secure stable institutional partnerships with healthcare organizations to ensure adequate resources for specialized first aid education activities.

### 4.4 Participants' and researchers' reflexivity

Participants' reflective statements throughout the interviews revealed sophisticated metacognitive awareness of their identity transformation process. Many students articulated explicit recognition of their evolving self-concept, describing moments when they realized they were no longer merely performing volunteer service for credits but had genuinely embraced their role as health educators. This reflexivity was particularly evident when students discussed their emotional responses to teaching challenges, with several noting how initial anxiety about making mistakes gradually transformed into confidence through repeated successful teaching experiences. Students' capacity to critically reflect on their own development, acknowledging both the influence of external motivators and their internal growth, demonstrates the depth of identity transformation facilitated by FAKPV participation. As researchers with backgrounds in medical education and volunteer service, we observed that this metacognitive awareness itself represents an advanced stage of professional development, suggesting that FAKPV not only promotes skill acquisition but cultivates the reflective capacity essential for lifelong professional learning.

### 4.5 Implications for medical education

Our findings demonstrate that FAKPV participation develops core competencies aligned with authoritative medical education frameworks (63). Teaching first aid to community members enacts health advocacy and addresses population-level community health needs, while translating medical knowledge for lay audiences hones communication skills—including plain language use, cultural sensitivity, and audience adaptation—critical to clinical and public health practice (64).

FAKPV also fosters professional values (altruism, social responsibility), leadership, and interprofessional collaboration through interdisciplinary teamwork and community partner coordination (65). Additionally, engaging with community health needs, mobilizing resources, and reflecting on teaching effectiveness to drive improvement cultivates systems thinking and lifelong learning—key pillars of modern medical education (66).

Notably, FAKPV could serve as an authentic assessment tool for competencies traditionally hard to evaluate via conventional methods (67). Medical schools should develop structured FAKPV programs with explicit competency mapping and standardized rubrics to document students' professional development across multiple domains simultaneously. Such integration of community-based volunteer education into curricula has been shown to enhance practical skills and professional identity (68).

### 4.6 Limitations

Our study has certain limitations. First, participants were recruited from a single medical university in Sichuan Province, China, and screened by authorized staff from the volunteer organization, which may have introduced gatekeeper bias by preferentially recommending students with positive volunteer experiences. Additionally, the Chinese cultural context may have influenced participants' volunteer motivations in ways that limit international applicability. Second, social desirability bias may have influenced participants to emphasize positive experiences and underreport negative aspects, particularly given that recruitment was facilitated through the volunteer organization. Third, our data collection relied primarily on interviews, which introduces subjective elements and lacks objective measurements from multiple stakeholder perspectives. Fourth, our cross-sectional design captured volunteer experiences at a single time point rather than tracking longitudinal trajectories. Fifth, while we employed investigator triangulation, the single-method qualitative approach may limit comprehensive understanding compared to mixed-methods designs. Future research could extend to diverse volunteer groups across various fields, use alternative recruitment strategies to minimize gatekeeper bias, employ mixed-methods approaches incorporating multiple data sources, and utilize additional theoretical frameworks to provide alternative analytical perspectives.

## 5 Conclusion

Our qualitative descriptive study, using Expectancy-Value Theory as the theoretical framework, conducted 27 semi-structured interviews with undergraduate medical students and analyzed data through Braun and Clarke's thematic analysis approach. Our findings reveal that undergraduate medical students participate in FAKPV primarily due to four motivations, including voluntary spirit, self-achievement, rewards, and peer influence. They face challenges, including skill gaps, psychological stress, logistical constraints, and time conflicts, while gaining psychological benefits, motivational incentives, and improved competencies. These findings provide insights into optimizing the model of medical education and medical student volunteer service. To enhance motivation, universities should develop specialized first aid education curricula integrating emergency simulation scenarios and establish FAKPV-specific peer mentorship programs. To address challenges, medical schools should provide specialized FAKPV training programs combining first aid techniques with public education pedagogy and establish partnerships with local emergency medical services for simulation experiences. To maximize benefits, FAKPV programs should create opportunities for volunteers to witness direct impact through follow-up assessments and establish competency recognition systems.

## Data Availability

The original contributions presented in the study are included in the article/Supplementary material, further inquiries can be directed to the corresponding authors.

## Supplementary material

Supplementary File 1 COREQ checklist.

Supplementary File 2 Interview guide.

Supplementary File 3 Coding examples.
