# Supplementary material for: Motivations, challenges, and benefits of first aid knowledge popularization volunteerism among undergraduate medical students: a qualitative study
Source: Front Public Health. 2025 Dec 31;13:1701431. doi: 10.3389/fpubh.2025.1701431 (PMC12801344; doi:10.3389/fpubh.2025.1701431)
Supplement: Supplementary File 1 — COREQ checklist. [file Table_1.docx]

COREQ Checklist

| **Item No. and Topic** | **Guide Questions/Description** | **Response and Reported on Page No.** |
| --- | --- | --- |
| **Domain 1: Research team and reﬂexivity** | | |
| *Personal characteristics* | | |
| 1. Interviewer/facilitator | Which author/s conducted the interview or focus group? | Siyu Li |
| 2. Credentials | What were the researcher’s credentials? E.g. PhD, MD | Siyu Li: Undergraduate Student  Ruiyu Huang: PhD  Yanxia Guo: PhD  Xiaofang Yang: Bachelor's degree  Baolu Zhang: PhD |
| 3. Occupation | What was their occupation at the time of the study? | Siyu Li: Undergraduate Student  Ruiyu Huang: Lecturer  Yanxia Guo: Lecturer  Xiaofang Yang: Attending physician  Baolu Zhang: Lecturer |
| 4. Gender | Was the researcher male or female? | Siyu Li: Female  Ruiyu Huang: Female  Yanxia Guo: Female  Xiaofang Yang:Female  Baolu Zhang: Female |
| 5. Experience and training | What experience or training did the researcher have? | Researchers have experience in volunteer service and qualitative interviews. |
| *Relationship with participants* | | |
| 6.Relationship established | Was a relationship established prior to study commencement? | Siyu Li screened participants through the first- aid knowledge popularization volunteer team. Prior to the study, the researchers had no relationships with the participants. p.6 |
| 7. Participant knowledge of the interviewer | What did the participants know about the researcher? e.g. personal goals, reasons for doing the research | Participants were aware of the reasons for agreeing to take part in the research study. The researcher had a phone conversation with the participants before the interview and obtained their consent.p.6 |
| 8.Interviewer characteristics | What characteristics were reported about the inter viewer/facilitator? e.g. Bias, assumptions, reasons and interests in the research topic | The interviewing researchers shared their names, research backgrounds, organisational affiliations, and background of the project to participants.p.6 |
| **Domain 2: Study design** | | |
| *Theoretical framework* | | |
| 9. Methodological orientation and Theory | What methodological orientation was stated to underpin the study? e.g. grounded theory, discourse analysis, ethnography, phenomenology, content analysis | Expectancy-value theory and thematic analysis.p3-4.p7-8 |
| *Participant selection* | | |
| 10. Sampling | How were participants selected? e.g. purposive, convenience, consecutive, snowball | Purposive.p.6 |
| 11. Method of approach | How were participants approached? e.g. face-to-face, telephone, mail, email | Telephone.p.6 |
| 12. Sample size | How many participants were in the study? | 27 first-aid knowledge popularization volunteer team members.p.5 |
| 13. Non-participation | How many people refused to participate or dropped out? Reasons? | None of the participants refused to participate or dropped out. |
| *Setting* | | |
| 14. Setting of data collection | Where was the data collected? e.g. home, clinic, workplace | Workplace.p6 |
| 15. Presence of non-participants | Was anyone else present besides the participants and researchers? | Only the participants and interviewers were present.p.6 |
| 16. Description of sample | What are the important characteristics of the sample? e.g. demographic data, date | Demographics of the study participants are outlined in Table 1 and Tcble 2.p.25-26 |
| *Data collection* | | |
| 17. Interview guide | Were questions, prompts, guides provided by the authors? Was it pilot tested? | The questions are provided by the author. It was not pilot tested.p.6 |
| 18. Repeat interviews | Were repeat interviews carried out? If yes, how many? | Repeat interviews were not carried out. |
| 19. Audio/visual recording | Did the research use audio or visual recording to collect the data? | The audio and video of interviews were recorded.p.6 |
| 20. Field notes | Were ﬁeld notes made during and/or after the interview or focus group? | Field notes were made immediately after each interview and aided in data analysis.p.6 |
| 21. Duration | What was the duration of the interviews or focus group? | 26-54 minutes.p.9 |
| 22. Data saturation | Was data saturation discussed? | Yes, data adequacy was discussed.p.8 |
| 23. Transcripts returned | Were transcripts returned to participants for comment and/or correction? | No |
| **Domain 3: Analysis and ﬁndings** | | |
| *Data analysis* | | |
| 24. Number of data coders | How many data coders coded the data? | Two researchers.p.7 |
| 25. Description of the coding tree | Did authors provide a description of the coding tree? | Table 1.p.24 |
| 26. Derivation of themes | Were themes identiﬁed in advance or derived from the data? | Themes were derived from the date.p.9-15 |
| 27. Software | What software, if applicable, was used to manage the data? | No |
| 28. Participant checking | Did participants provide feedback on the ﬁndings? | No |
| *Reporting* | | |
| 29. Quotations presented | Were participant quotations presented to illustrate the themes/ﬁndings? Was each quotation identiﬁed? e.g. participant number | Yes, participant quotations were presented with numbers.p.9-15 |
| 30. Data and ﬁndings consistent | Was there consistency between the data presented and the ﬁndings? | Yes, as indicated in the discussion.p17-23 |
| 31. Clarity of major themes | Were major themes clearly presented in the ﬁndings? | Yes, presented in Results.p9-15 |
| 32. Clarity of minor themes | Is there a description of diverse cases or discussion of minor themes? | Yes, presented in discussion.p17-23 |
