# Supplementary material for: Motivations, challenges, and benefits of first aid knowledge popularization volunteerism among undergraduate medical students: a qualitative study
Source: Front Public Health. 2025 Dec 31;13:1701431. doi: 10.3389/fpubh.2025.1701431 (PMC12801344; doi:10.3389/fpubh.2025.1701431)
Supplement: Supplementary File 2 — Interview guide. [file Table_2.docx]

Supplementary File: Semi-Structured Interview Guide

**Study Title:** Motivations, Challenges, and Benefits of First Aid Knowledge Popularization Volunteer Activities Among Undergraduate Medical Students: A Qualitative Study Applying Expectancy-Value Theory

**Introduction Script**

Thank you for agreeing to participate in this research. I would like to know about your experience in the FAKPV activities. There are no right or wrong answers - what interests me is your personal experience and perspective. With your permission, I will record our conversation. Everything you share will be kept confidential. You can choose not to answer any questions or stop the interview at any time. Before we begin, do you have any questions?

**Section 1: Background and Participation**

1. Can you start by telling me about yourself and your involvement with FAKPV?

Probes:

How long have you been participating?

What types of FAKPV activities have you participated in?

How many times have you participated?

2. How did you first learn about FAKPV?

Probes:

What made you decide to join?

Did anyone influence your decision?

3. What motivated you to initially join FAKPV activities?

Probes:

What were you hoping to gain?

Were there any specific reasons or goals?

Did external factors (credits, requirements) play a role?

4. What keeps you involved in FAKPV activities now?

Probes:

What aspects do you find most meaningful?

5. How do you feel about the concept of volunteering and helping others through FAKPV?

Probes:

Do you see this as connected to your identity as a medical student?

How does it relate to your future career goals?

6. Have friends or classmates influenced your participation?

Probes:

Did you join with peers?

How do others view FAKPV participation?

7. What challenges or difficulties have you encountered during FAKPV participation?

Probes:

Can you give me specific examples?

Were there situations where you felt unprepared?

8. How confident do you feel teaching first aid to the public?

Probes:

Have you experienced any anxiety or nervousness?

What situations make you feel uncertain?

How adequate is your knowledge and skill level?

9. How do you balance FAKPV activities with your academic responsibilities?

Probes:

Have you experienced time conflicts?

How do you manage your schedule?

10. Are there any external barriers that affect your participation?

Probes:

Resource limitations?

Organizational challenges?

11. What have you gained from participating in FAKPV activities?

Probes:

Personal growth?

Skill development?

12. Have you received any tangible benefits from participation?

Probes:

Academic credits or recognition?

Career development opportunities?

Practical applications of learned skills?

13. How do you feel after completing FAKPV activities?

Probes:

Sense of accomplishment?

Fulfillment?

**Section 2: Overall Experience and Reflection**

14. Looking back on your FAKPV experience, what stands out most to you?

Probes:

Most memorable moments?

Most significant learning?

15. Would you recommend FAKPV to other medical students? Why or why not?

16. Is there anything else about your FAKPV experience that you would like to share?

**Closing**

Thank you so much for sharing your experiences with me. Your insights are very valuable for understanding how medical students experience volunteer teaching activities. Do you have any questions for me about the study?
